# The effects of narrative care combined with life review on psychological distress, meaning in life, and quality of life in advanced cancer patients receiving hospice care: a randomized clinical trial

**DOI:** 10.3389/fpsyg.2025.1656772

**Published:** 2025-12-02

**Authors:** Juan Wang, Yuan Xia, Qinqin Cheng, Ling Chen

**Affiliations:** 1 The Affiliated Cancer Hospital of Xinjiang Medical University, Ürümqi, China; 2 Linyi People’s Hospital, Linyi, China; 3 The Affiliated Cancer Hospital of Xiangya School of Medicine, Central South University/Hunan Cancer Hospital, Changsha, China

**Keywords:** narrative care, life review, hospice, patients with advanced cancer, randomized clinical trial

## Abstract

**Objective:**

To investigate the effects of narrative care combined with life review among patients with advanced cancer.

**Methods:**

Ninety patients who met the inclusion criteria were recruited from the palliative medicine ward of a tertiary-level oncology specialty hospital in Xinjiang and the community hospitals hosted by our hospital from January 2024 to January 2025. This study is a randomized controlled single-blind study. They were randomly divided into control and experimental groups (*n* = 45 patients per group). The control group received usual care, whereas the experimental group received narrative nursing combined with life review as a psychological intervention in addition to the usual care received by the control group. The number of interventions per patient was not less than 4 times, and the intervention period was 2 weeks. Before and after the intervention, the experimental and control groups were evaluated using the Psychological Distress Management Screening Tool, the Sense of Meaning of Life Scale for Advanced Cancer Patients, and the EORTC Quality of Life Measurement Scale QLQ-C30.

**Results:**

Eighty-three participants completed the study. No statistically significant differences were observed in psychological distress, scores for the total meaning of life scale and its individual dimensions, and scores for the total quality of life scale and its individual dimensions between the two groups before the intervention (*p* > 0.05). After the intervention, the distress thermometer scores were significantly lower in the experimental group than in the control group (*p* < 0.05). The scores for the total meaning of life scale and its individual dimensions were significantly higher in the experimental group than in the control group (*p* < 0.05). The scores for the total quality of life scale and its individual dimensions were significantly higher in the experimental group than in the control group (*p* < 0.05).

**Conclusion:**

Narrative nursing combined with life review can effectively alleviate the psychological suffering, increase the sense of meaning of life to a certain extent, and improve the quality of survival among patients with advanced cancer. It is recommended to incorporate it into routine hospice care practice, through structured life review guidance, to help patients affirm their self-worth and enhance their dignity and calm at the end of life.

## 1 Introduction

Cancer is a malignant disease caused by gene mutations that cause abnormal cell proliferation and may metastasize. It imposes a multi-dimensional burden on patients: physically causing pain, weakness and impaired function; mentally trigger fear, anxiety and depression; at the social level, it brings economic pressure, tension and social isolation, which comprehensively affects the quality of life of individuals. According to the latest Global Cancer Report 2020 released by the World Health Organization (WHO) (Zheng et al., 2023), the number of new cancer cases worldwide was 18.1 million in 2018, and it is estimated to increase by 60% in the next 20 years. In 2018, China had the leading numbers of new cancer cases and cancer-related deaths at 3.804 million and 2.296 million, respectively. Cancer has been the leading cause of death in China, and its incidence rate, mortality rate, and burden are all increasing (Qi et al., 2023). Hospice care is a type of palliative care for patients in the last 6 months of life (Shalev et al., 2018), a systematic approach offered to patients and families with life-threatening illnesses to improve their quality of life and ability to cope with crises. Through the early identification of distress and pain, rigorous assessment, and effective management, all of the psychological and spiritual needs of the patient and their family can be met. Some studies have pointed out that the demand for hospice care is the highest among cancer patients in China (Lyerly et al., 2015). With the advances and improvements in medical services, people’s understanding of disease and health has changed, and the medical model has gradually transformed from the traditional medical model consisting of “pathological–physiological” aspects to a new medical model of the “physiological–psychological–social” aspects of health (Ma et al., 2024). Compared with the traditional medical model, the new medical model pays more attention to the patient’s psychological and social factors and regards disease as the response of the body to the joint effect of the two types of factors on the human body. Under the new medical model, humanistic medical care is receiving increasing attention. High-quality medical care involves not only relieving the patients’ pain and symptoms but also enhancing their experience of and satisfaction with medical treatment.

Life review is a process in which participants review (Huang et al., 2020), evaluate, and summarize their life experiences under the interventionist’s structured guidance, discover unresolved conflicts in their lives, analyze and reorganize these conflicts, recall the good memories of the past, and reconcile with the past, all to improve their state of mind and discover new meaning in their lives. After learning the method, patients can engage in independent, ongoing practice through methods like journaling or creating a life storybook to continuously integrate new experiences and enhance psychological resilience. Its theoretical foundation stems from Erikson’s Stages of Psychosocial Development (Haber, 2006). It was formally proposed by psychiatrist Robert Butler in the 1960s and has since evolved into an effective method widely used to enhance mental health and sense of meaning in life. Life review is mainly adopted for patients with advanced cancer, those receiving hospice care (Wang et al., 2017), those living with illness (Subramaniam et al., 2014), and older adults (Al-Ghafri et al., 2021) to enhance their sense of dignity and well-being (Lan et al., 2018), reduce their self-burden (Tam et al., 2021), alleviate their fear of death (Xiao et al., 2013), and improve their quality of life. Narrative nursing (Hairong, 2017) is a model of nursing practice in which the caregiver witnesses, understands, experiences, and responds to the patient’s distressing condition with narrative competence, including love, acceptance, and compassionate care, and provides comfort and relief to the patient. Narrative nursing as a model of psychological care to be developed by Professor White and Epston (1990), based on the proposed Narrative Therapy, Professor Sandelowski (1994) first proposed Narrative Nursing. Tt is mainly used in the context of psychosocial rehabilitation for special populations, adolescents (Gong et al., 2022), female patients (Jin et al., 2022), psychiatric patients (Zhou et al., 2023), cancer patients and their caregivers (Wang et al., 2024), and a variety of other populations, including the caregivers themselves (Fitzpatrick, 2021), as well as in different therapeutic contexts such as in the community, university counseling centers, and hospitals. The main subjects of implementation are doctors, nurses, psychotherapists, etc. Narrative nursing forms include face-to-face thematic interviews, watching other people ‘s narrative works, making digital stories, and disease narratives based on the Internet (Cleary and Stanton, 2015). Currently, narrative care is integrated with other therapies in clinical psychology in China and abroad to address psychosocial problems such as anxiety and depression (Jalali et al., 2019), schizophrenia (White and Epston, 1990), affective disorders (Focht and Beardslee, 1996), and post-traumatic stress disorder (Wigard et al., 2024). In the contexts of outpatient and inpatient treatment, narrative care can alleviate patients’ negative emotions (Wang et al., 2023), rebuild their meaning of life, and facilitate their treatment in rehabilitation (Liu et al., 2025). Life review can help patients to review, evaluate, reorganize, and dissect their life experiences. This study is the first to combine narrative nursing and life review to help patients link their past and present, organize and express their emotions, reexamine and share their life experiences, meet their psychological and social support needs, and rethink the meaning of life in a meaningful exploration.

Psychological distress, quality of life, and meaning in life are core dimensions for measuring an individual’s mental health and overall well-being. Psychological distress is an unpleasant emotional and affective state, typically arising when an individual perceives themselves as unable to effectively cope with internal or external stressors. Meaning in life refers to an individual’s perception and understanding of the value, purpose, and significance of their existence. Quality of life is a subjective perception and evaluation of one’s position and level of satisfaction across various domains of life, emphasizing the overall “goodness” or “badness” of living conditions. By exploring the effects of narrative nursing combined with life review on psychological pain, life meaning and quality of life of patients with advanced cancer hospice care, the aim is to construct a hospice care model for patients with advanced cancer suitable for China ‘s national conditions and effectively improve the quality of life of patients and their families.

## 2 Materials and methods

### 2.1 Study design

A randomized controlled single-blinded design was adopted. This study is only for patients. This study was approved by the hospital’s ethical committee (approval number K2023043). The study procedure followed the principles of the Declaration of Helsinki. The study was reported in accordance with the CONSORT statement.

### 2.2 Participants

Advanced cancer patients who met the inclusion and exclusion criteria were recruited from the palliative medicine ward of a tertiary-level oncology specialty hospital in Xinjiang and the community hospitals. The inclusion criteria were as follows: patients (1) with a pathological diagnosis of advanced cancer, expected survival < 6 months, a hospitalization duration > 5 days, and a psychological distress (distress thermometer) score ≥ 4, as assessed by two doctors; (2) who had given up on treatment for the primary cancer; (3) who were willing to accept hospice care and sign an informed consent form; and (4) who can clearly express their feelings. The exclusion criteria were as follows: patients who were participating in a similar study. Informed consent was obtained from all participants.

### 2.3 Sample size estimation

The psychological distress was assessed as the primary outcome and the sample size was calculated using the formula n1 = n2 = 2[(Z_α_ + Z_β_)*δ*/d]^2^. According to the literature (Xu et al., 2021), for a standard deviation *δ* of 1.38 and a difference *d* of 1.3 between the means of the two groups, at least 45 participants are required in each group, accounting for a 20% dropout rate. Thus, a total of 90 patients were considered to be required for this study. A member who was not involved in the intervention study was numbered according to the patient ‘s admission time sequence and imported into SPSS to generate random numbers. The 90 patients were randomly divided into the control group (*n* = 45) and the experimental group (*n* = 45) using the random number table method. A total of 83 patients completed the study, participants with missing data were removed, this constitutes a complete-case analysis. To avoid contamination, the patients in the experimental group were asked not to communicate with the control group about the content of the intervention.

### 2.4 Intervention

#### 2.4.1 Control group

The control group received routine care following the “Hospice Practice Guidelines (Trial)” as the working guideline (The National Health and Family Planning Commission of the People’s Republic of China, 2017). According to this guideline, the nursing routine of the department is formulated to address 13 items of symptom control and 16 items of comfort care, and it involves giving routine hospice care as well as routine psychological care and health education guidance during the hospitalization period, including educating the patients on disease-related knowledge, diet, activities, and infection prevention; educating and psychologically comforting the main caregivers; and instructing them to care for and encourage the patients.

#### 2.4.2 Experimental group

The narrative care combined with life review team consisted of seven members: the head nurse of the ward, a second-level psychological counselor, four national hospice specialist nurses, and a nursing graduate student. The team members divided their work. The author of the communication is responsible for the design of the implementation plan. The first author is a national secondary psychological counselor, responsible for the implementation plan. The second author is a nursing graduate student, responsible for data collection and data analysis. All authors received 2 weeks of relevant training.

The experimental group received the narrative care combined with life review intervention in addition to the usual care received by the control group. The team used listening, empathy, and other skills to maintain verbal or nonverbal communication with the patients. This was carried out in the department’s spiritual communication room, which offered a quiet and comfortable environment to protect privacy, avoid interrupting or terminating the patient’s thoughts and ideas, and enable them to fully express their feelings. The intervention content was formulated by consulting relevant literature (Lin et al., 2024), and the intervention plan was determined by expert consultation. The intervention comprised four phases: the implementation of the interactive stage of concern, life review stage, narrative construction stage, and response to the end of the stage. The intervention comprised four face-to-face interview sessions of 30–45 min each, starting from the first day of the patient’s hospitalization and every 2 to 3 days thereafter. The intervention period was 2 weeks and the content is shown in Table 1.

**Table 1:** Narrative care combined with life review intervention.

Procedure	Time	Theme	Intervention content	Specific implementation
Stage 1	1–2 days	Relationship interaction	1. Build a trusting relationship: Through open communication and listening, build a good relationship with patients and make them feel understood and cared for. 2. Ask about their emotional state: Understand the patient’s current emotional state, including anxiety, fear, and loneliness, to prepare for the subsequent life review process. 3. Confirm their willingness to participate: Ensure that the patients are willing to participate in the life review process and understand their expectations and needs.	First, a quiet and comfortable environment was selected for the intervention. The team members and the patients introduced themselves, and the team members listened to establish a trust relationship with the patients. They used positive language to encourage the patients to express their concerns and understanding and used open-ended questions to guide the conversation, such as “Please share some life experiences that you find important or meaningful to you.” This helped to initiate topics and communicate with patients about the goals and expectations of a life review, such as helping them understand past experiences, promoting emotional processing, or reducing psychological burden. Critical information shared by the patients at this stage were appropriately documented for the subsequent life review phase. The documentation was conducted with care and sensitivity to avoid disturbing the patient’s emotional expression and privacy. Interview outline: What is the most important thing in your life? What are the definitions and expectations of quality of life? What are your preferences and goals for medical care and quality of life? What role does family and intimacy play in your life? Is there any particular information or decision that you want to share with your family or friends?
Stage 2	3–7 days	Life review	1. Guide the review process: Help patients recall important moments and experiences of their life by asking questions or leading the conversation. 2. Record key information: Record important memories and stories shared by patients, which can help to understand the patients’ outlook on life and values. 3. Support emotional expression: During the review process, support the patients to express complex emotions, including reflecting on challenges and achievements in life.	The team members helped patients to recall vital moments, events, and people in their lives by asking questions and providing guidance, and helped them review vital life events by providing timelines, family dendrograms, or other visualization tools. During the guided review process, team members actively recorded important information and stories that the patients were willing to share. The conversations were audiotaped or noted down to ensure that no important details or feelings were omitted. During the review process, patients may express complex emotions, such as regret, pride, and gratitude. The team members supported and accepted these emotional expressions through appropriate responses to encourage the patients’ emotional release and integration. The team members explored in depth the key topics expressed by the patients to help them more clearly understand their meaning and value in life. This involved the exploration of the meaning of life, the importance of relationships, and regrets. To ensure that the patients had enough time and space to think and express, the life review process of each patient was individualized to understand and respect individual differences. Interview outline: Review the most important stages or events of your life. How have these experiences affected your growth and life concept? What are you most proud of or satisfied with? Are there any regrets or unfulfilled wishes in your life?
Stage 3	8–12 days	Narrative construction	1. Integration and interpretation: Help patients to integrate memories into an orderly narrative that explains the ups and downs and turning points in their lives. 2. Strengthen personal meaning: Emphasize achievements and personal growth in life and help patients recognize their unique meaning and value in life.	The team members discussed with the patients to identify and clarify the problems that needed to be addressed. In this process, the team members respected the patients’ opinions and feelings that the problem was real. They helped to transform problems from abstract descriptions to concrete images or situations to better understand and solve them. They helped externalize the problem by guiding the patients to describe the problem using props or pictures. At this stage, the team members made efforts to deeply understand the background and causes of the problem, including the patient’s living environment, experience, and psychological state. This facilitated a more comprehensive understanding of the problem and the search for suitable solution strategies. The team members helped the patients to reconstruct cognition and understand their problems by guiding them to look at the problems from different perspectives, thus helping them to understand the nature and impact of their problems. This helped the patients to release negative emotions and find positive solutions. The team worked with the patients to develop clear goals and plans to address the externalized issues. The objectives were specific and feasible, and the plan included specific steps and time arrangement. They assisted the patients in identifying and exploring themes and values in life, such as family, relationships, and achievements. Integrating emerging material from a life review into the narrative helped them to see how past challenges had shaped their self-identity and supported positive self-discovery and acceptance. Interview outline: What is the main message that you want to convey to others through your life story? Is there a specific story or event that you feel is very important? Are there any special characters or scenes that need to be highlighted? Who would you like to listen to your story?
Stage 4	14th day	Response to the end of life	Summary and reflection: Review the whole process with patients and summarize important insights and feelings. 2. Provide support and comfort: Communicate support and comfort to patients to ensure that they feel understood and respected. 3. Make a future plan: Discuss possible follow-up actions or support measures to help patients move on in life.	After completing the life review and narrative construction, the team members evaluated the effects of narrative care, including understanding the changes in the patients’ perspectives on their life story, their emotional improvement, and the improvement in their life quality. The team members continued to provide support and assistance to the patients, including supporting emotional expression and handling of unfulfilled life wishes, unresolved relationship problems, or other regrets. They helped the patients find emotional release and comfort through listening and providing support. In addition, regular follow-up and evaluation were conducted to monitor the patients’ progress and changes. Finally, the team members summarized and reflected on the entire intervention process, summarized the lessons learned, and analyzed successes and shortcomings to further improve the quality of narrative care. Interview outline: What are your special expectations or needs for your last days? Are there any special requirements for comfort and pain management? Do you need special family meetings or assistance from a social worker? What is your current emotional state? Do you have a special psychological or spiritual support need?

The team members received standardized training to ensure the consistency of the implementation of the intervention and usual care. They learned the relevant knowledge to reduce the risk of bias, strictly followed the inclusion and exclusion criteria, and were instructed not to select research subjects based on any subjective reasons to ensure the scientific nature and authenticity of the research.

### 2.5 Measurements

#### 2.5.1 General information questionnaire

The self-designed general information questionnaire mainly covered age, gender, marital status, education level, family income, and residence type.

#### 2.5.2 Distress management screening measure

The Distress Management Screening Measure, developed by Zhang et al. (2010). in 2010, was used to measure the degree and source of psychological distress in our advanced cancer patients. It comprises two parts: the Distress Thermometer (DT) and the Problem List (PL). The DT is a one-item screening tool that measures the degree of psychological distress using numbers. The PL lists 40 specific problems across five aspects, including practical problems, communication problems, emotional problems, and physical problems. The scale has a Cronbach’s *α* coefficient of 0.75.

#### 2.5.3 Meaning of life in patients with advanced cancer scale

The Meaning of Life in Patients with Advanced Cancer Scale developed by Yongsheng and Jincai (2009) was used to evaluate the meaning of life perceived by our advanced cancer patients. The scale consists of 28 items across six dimensions (the will to seek meaning, frustration, life meaning and satisfaction, life control, suffering, and death acceptance). Each item is scored on a 5-point Likert scale from 1 to 5: 1 = “very inconsistent”; 2 = “basically inconsistent”; 3 = “neutral or uncertain”; 4 = “basically consistent”; and 5 = “very consistent.” Reverse entries were scored reversely, with the total score ranging from 28 to 140. A higher score indicates a higher meaning of life. The Cronbach’s *α* coefficient for this scale is 0.725.

#### 2.5.4 EORTC quality of life measurement scale QLQ-C30

The Chinese version of the Quality of Life Core Scale developed by the European Organization for Research and Treatment of Cancer and translated by Wan et al. (2005) was used. The scale consists of 30 items that assess five aspects of overall health status, functional dimensions, symptom dimensions, and the impact of disease on individual economy. The Cronbach’s *α* values for the individual dimensions of this scale range from 0.648 to 0.865 with retest reliability values of 0.611 to 0.843. The Cronbach’s α coefficient of the total scale is 0.798.

### 2.6 Data collection

Nurses and graduate students who were not involved in the intervention collected the questionnaires before and after the intervention, checked each returned questionnaire for completion, eliminated those with missing data, wrong data, or obvious answers, and checked the input data to ensure data accuracy.

### 2.7 Statistical processing

Statistical analyses were conducted using the data processing software SPSS 26.0 and the mapping software GraphPadPrism7. Count data are described as case numbers and composition ratios. The chi-square test and one-tailed independent-samples t-test were used for comparison between groups as appropriate, and the results are described as mean ± standard deviation. Grade data were tested using the rank sum test, with the test level *α* set at 0.05. The Shapiro–Wilk test was used to assess the normality of the data, and the data of each group followed a normal distribution (*p* > 0.05). Missing data were deleted (Heng et al., 2019). Differences were considered statistically significant at *p* < 0.05.

## 3 Results

**Figure 1:**
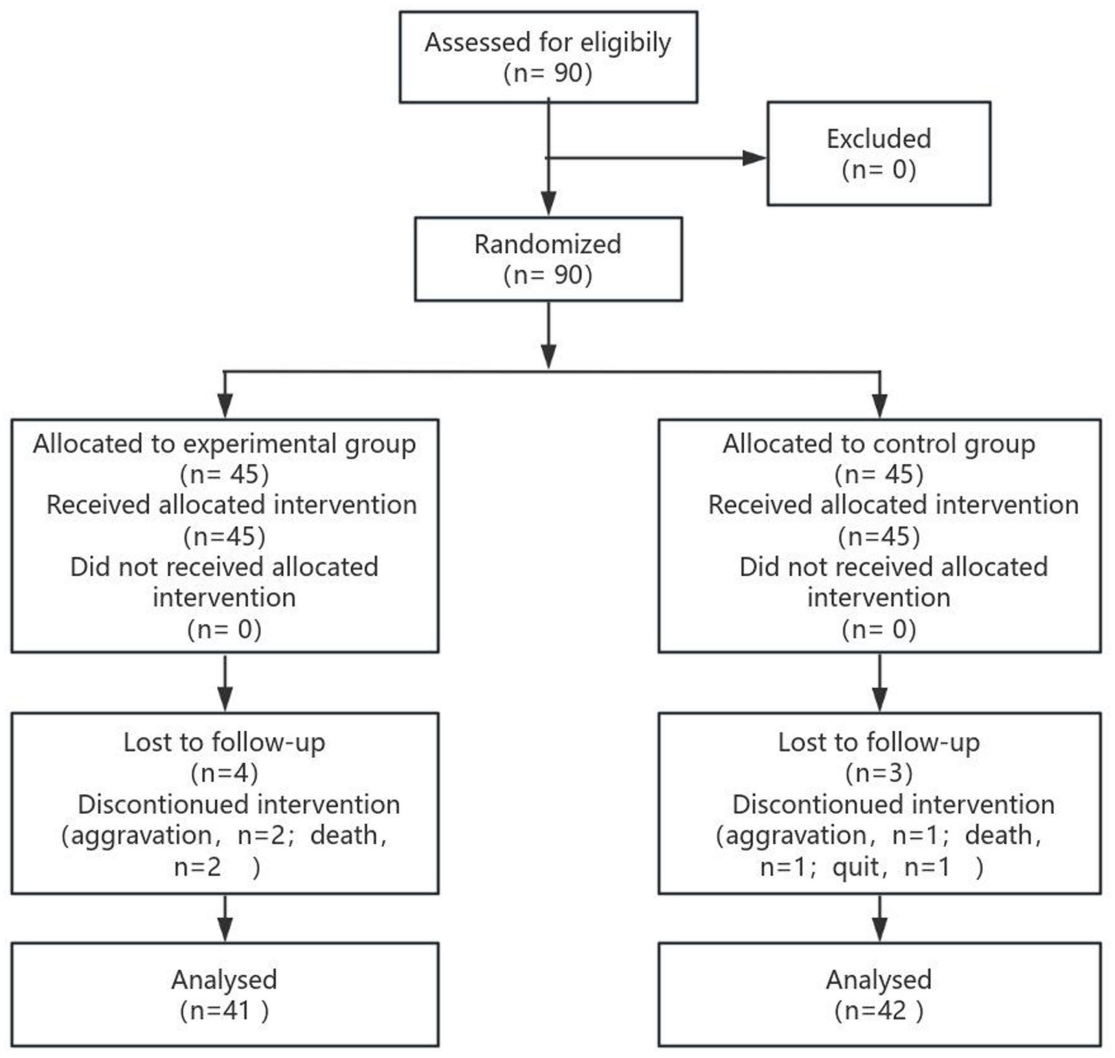
Flow chart for this study.

From January to September 2023, 90 patients, 45 in each group, were included in the study. In the experimental group, four patients dropped out: the condition of two patients worsened, and two patients died during the second week of the intervention; thus, 41 patients in this group completed the study. In the control group, three patients dropped out: the condition of one patient worsened during the first week of the intervention, and the condition of one more patient worsened and one patient withdrew during the second week of the intervention; thus, 42 patients in this group completed the study. The patient flow chart is shown in Figure 1.

### 3.1 Comparison of the general characteristics between the two groups

The general characteristics of the patients were not significantly different between the two groups (*p* > 0.05; Table 2).

**Table 2:** General characteristics of patients in the two groups.

Group	Experimental group (*n* = 41)	Control group (*n* = 42)	χ^2^/t	*P*
Age (year, *x ± s*)	61.49 ± 15.83	60.40 ± 15.57	0.314^c^	0.754
Gender (%)				
Male	16 (9.0)	17 (40.5)	0.018^a^	0.893
Female	25 (61.0)	25 (59.5)		
Marital status (%)				
Married	34 (82.9)	33 (78.6)	2.337^a^	0.311
Unmarried	4 (9.8)	8 (19.0)		
Others	3 (7.3)	1 (2.4)		
Education level (%)				
Junior high school and below	17 (41.5)	14 (33.3)	−0.992^b^	0.321
High school/technical secondary school	16 (39.0)	16 (38.1)		
College degree or above	8 (19.5)	12 (28.6)		
Family monthly household income (Yuan, %)				
< 3,000	4 (9.8)	3 (7.1)	−0.088^b^	0.930
3,000–5,000	23 (56.1)	25 (59.5)		
> 5,000	14 (34.1)	14 (33.3)		
Whether the family can financially afford the treatment of the disease (%)				
Completely	10 (24.4)	11 (26.2)	−0.478^b^	0.633
Barely	23 (56.1)	25 (59.5)		
With difficulty	8 (19.5)	6 (14.3)		
Extended family	4 (9.8)	4 (17.0)	1.050^a^	0.690
Living with the children/parents	14 (34.1)	15 (22.6)		
Grandparents and grandchildren live together	0 (0.0)	1 (2.4)		
Husband and wife live together	19 (46.3)	18 (52.8)		
Live alone	4 (9.8)	4 (7.5)		
Primary caregiver during the hospital stay (%)				
Spouse	18 (43.9)	19 (45.2)	1.490^a^	0.828
Parents/children	17 (41.5)	16 (38.1)		
Another blood relative	2 (4.9)	1 (2.4)		
Friend	0 (0.0)	1 (2.4)		
Nursing assistant	4 (9.8)	5 (11.9)		
Cancer stage (%)				
III	25 (61.0)	25 (59.5)	1.018^a^	0.893
IV	16 (39.0)	17 (40.5)		
Cancer type (%)				
Lung cancer	6 (14.6)	8 (19.0)	1.217^a^	0.943
Liver cancer	4 (9.8)	5 (11.9)		
Gastric cancer	7 (17.1)	9 (21.4)		
Colorectal cancer	6 (14.6)	4 (9.5)		
Breast cancer	6 (14.6)	6 (14.3)		
Others	12 (29.3)	10 (23.8)		

^a^is the chi-square test; ^b^is the rank sum test; ^c^is *t* test.

### 3.2 Comparison of psychological distress between the two groups

**Figure 2:**
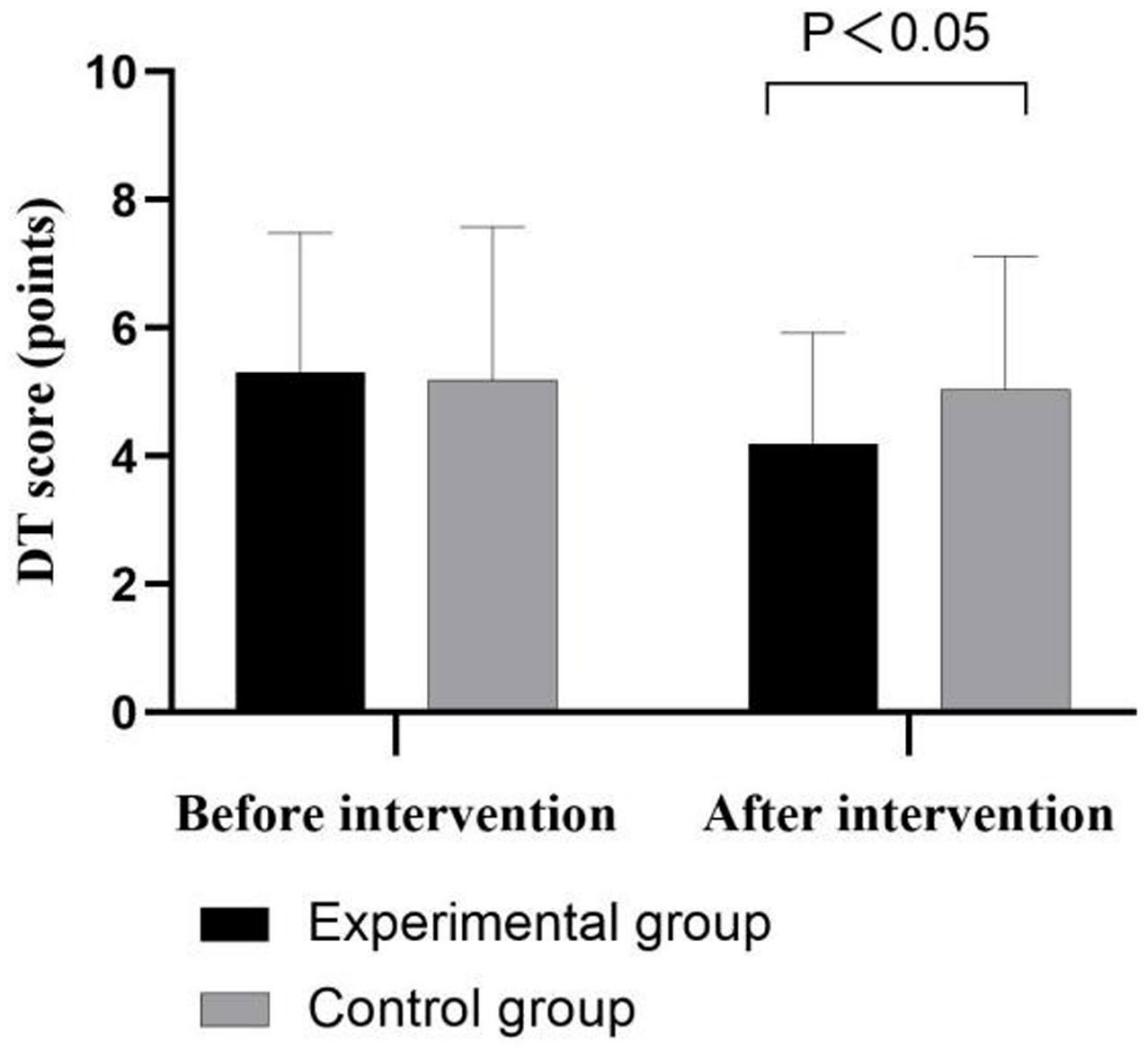
Comparison of psychological distress before and after the intervention between the two groups.

No significant difference was found in the baseline DT scores between the experimental and control groups (5.31 ± 2.18 vs. 5.19 ± 2.39, *t* = 0.480, *p* > 0.05); however, the post-intervention DT score was significantly lower in the experimental group than in the control group (4.19 ± 1.74 vs. 5.04 ± 2.08, *t* = 0.209, *p* < 0.05; Figure 2).

### 3.3 Comparison of meaning of life between the two groups

No significant difference was observed in the score of meaning of life between the two groups at baseline (*p* > 0.05) Experimental group and Control group The will to seek meaning(13.59 ± 3.44 *vs* 13.86 ± 3.24, *t* = −0.371, *p* = 0.712); Acceptance of setbacks(15.98 ± 2.77 vs 15.79 ± 3.02; *t* = 0.298, *p* = 0.766); Meaning of life(13.00 ± 2.57 vs 13.31 ± 2.63; *t* = −0.543, *p* = 0.589); Life control(23.95 ± 2.23 vs 24.02 ± 4.58; *t* = −0.067, *p* = 0.947); Suffering to bear(13.29 ± 3.09 vs 13.31 ± 2.68; *t* = −0.027, *p* = 0.979); Death acceptance(12.66 ± 2.86 vs 12.74 ± 3.00; *t* = −0.124, *p* = 0.902).

After the intervention, the meaning of life score was significantly higher in the experimental group than in the control group (*p* < 0.05; Table 3).

**Table 3:** Comparison of meaning of life between the two groups (mean ± SD).

Group	The will to seek meaning	Acceptance of setbacks	Meaning of life	Life control	Suffering to bear	Death acceptance
Experimental group (*n* = 41)	15.66 ± 2.04	17.85 ± 2.42	15.54 ± 1.95	26.22 ± 3.35	15.49 ± 2.05	14.51 ± 2.01
Control group (*n* = 42)	13.95 ± 2.53	16.24 ± 2.30	13.50 ± 2.13	24.38 ± 4.60	13.52 ± 2.62	13.26 ± 2.56
*t*	3.376	3.113	4.536	2.077	3.792	2.471
*p*	0.001	0.003	< 0.001	0.041	< 0.001	0.016

### 3.4 Comparison of quality of life between the two groups

No significant difference was observed in the quality of life scores between the two groups at baseline (*p* > 0.05). Experimental group and Control group Somatic function(45.69 ± 35.73 vs 46.67 ± 34.37; *t* = −0.127, *p* = 0.899); Role function(51.63 ± 38.69 vs 53.97 ± 33.70; *t* = −0.294, *p* = 0.769); Emotional function(62.80 ± 24.09 vs 63.49 ± 22.08; *t* = −0.136, *p* = 0.893); Cognitive function(54.88 ± 28.20 vs 56.35 ± 26.01; *t* = −0.247, *p* = 0.805); Social function(49.19 ± 32.13 vs 51.98 ± 32.13; *t* = −0.393, *p* = 0.695); Symptom(44.10 ± 21.27 vs 43.78 ± 18.16; *t* = 0.073, *p* = 0.942); Overall health status (109.35 ± 42.01 vs 106.75 ± 38.27; *t* = 0.295, *p* = 0.769).

After the intervention, the quality of life scores were significantly higher in the experimental group than in the control group (*p* < 0.05; Table 4).

**Table 4:** Comparison of quality of life between the two groups post-intervention (mean ± SD).

Group	Somatic function	Role function	Emotional function	Cognitive function	Social function	Symptom	Overall health status
Experimental group (*n* = 41)	60.98 ± 23.46	69.92 ± 20.82	72.76 ± 17.58	68.29 ± 21.35	67.48 ± 21.39	37.09 ± 15.58	127.23 ± 28.08
Control group (*n* = 42)	46.20 ± 29.46	54.37 ± 29.23	63.10 ± 20.34	55.16 ± 24.28	53.17 ± 30.41	46.10 ± 18.92	108.33 ± 34.58
*t*	2.532	2.797	2.315	2.619	2.483	−2.364	2.730
*p*	0.013	0.007	0.023	0.011	0.015	0.020	0.008

## 4 Discussion

The results of this study showed that the post-intervention DT scores were significantly lower in the experimental group than in the control group (*p* < 0.05, Figure 1), indicating that as a psychological care method, narrative care combined with life review intervention successfully reduced psychological distress by helping patients review their life experiences. In the face of end-stage psychological challenges, patients may experience a variety of emotions, including anger, depression, and anxiety (Song et al., 2013). Narrative care combined with life review enables patients to review their life processes by providing them with an emotionally safe environment in which to share their stories and experiences. This sharing not only facilitates the release of emotions and emotional integration but also reduces anxiety and depression, thus improving the mental health of patients. Zhang et al. (2023) conducted semi-structured in-depth interviews with advanced lung cancer patients undergoing chemotherapy who had received 2 months of narrative care and found that the implementation of narrative care promoted the patients’ growth in four psychological dimensions and relieved their psychological distress. In the face of advanced cancer and the inevitable death, patients often feel afraid and uneasy about the future (Curran et al., 2020). The implementation of narrative care combined with life review can help patients better accept the natural process of life and look for positive factors in life, thus reducing their fear of and anxiety regarding death and enhancing their sense of satisfaction with and acceptance of life. As a psychological nursing tool for psychological distress, narrative nursing combined with life review promotes patients’ psychological growth, emotional release, and life acceptance by providing them with a strong psychological support and help so that they can face the end of life with more dignity and peace.

The study results indicate that the scores of the experimental group was significantly higher than the control group (*p* < 0.05; Table 2), indicating that the narrative care combined with life review intervention had a profound impact on promoting the exploration and reconstruction of meaning of life among advanced cancer patients. The narrative care combined with life review intervention allows patients to review and reflect on critical moments, important relationships, and achievements in life, prompting them to reexamine and understand their lives, a process that can help patients establish a deep sense of self-identity. By constructing positive life stories, the intervention helps patients to understand their own existence and experiences from different perspectives and to discover the meaning and value in daily life. Positive narration emphasizing the internal resources of individuals helps them to face the challenges of treatment and life more actively so that they can face the end of life more calmly (Miao and Gan, 2020). Studies have shown (Ng et al., 2022) that the life review intervention for advanced cancer patients can reduce their psychological distress, improve their sense of life meaning, and enhance their bearing capacity. By sharing life stories with family, friends, and caregivers, patients are able to deepen and reinforce emotional connections with others. This type of sharing can not only enhance the understanding and support of the family and society but also help patients develop a deeper emotional legacy, enabling them to face the end of life in a more active and profound way and to experience a more meaningful and satisfying end of life (Ji et al., 2020).

Furthermore, the results demonstrated that the scores for the total quality of life scale and its individual dimensions were higher in the experimental group than in the control group post-intervention (*p* < 0.05; Table 3), indicating that the narrative care combined with life review intervention emphasized important experiences, promoted emotional connection and understanding between patients and society, and helped the patients to re-examine their identity and the significance of life. This process not only provided emotional warmth and support for the patients but also enhanced their self-identity and self-esteem, thereby improving their life satisfaction, quality of life, and resilience (Cepeda et al., 2008). The intervention also helped the patients to achieve a clearer understanding of their values and the significance of life, thus providing support and a basis for the development of personalized medical and hospice care plans. This in turn helped the patients to complete their life journey with more dignity. Thus, narrative nursing combined with life review not only supported advanced cancer patients psychologically and helped them rebuild their life meaning and identity but also provided them with strong support and help at the emotional, social, and physical levels, thus significantly improving their quality of life.

## 5 Conclusion

This study found that narrative nursing combined with life review effectively relieved patients’ psychological distress, enhanced their sense of meaning of life, and improved their quality of life. However, the treatment process requires personalized adjustments and guidance according to the specific situation of each patient. Due to the rapid change of the patient ‘s condition, no follow-up was conducted after the intervention, and further study is warranted to evaluate the long-term effects of the intervention so that narrative care can be appropriately adjusted in the later stage.

## Data Availability

The original contributions presented in the study are included in the article/supplementary material, further inquiries can be directed to the corresponding author/s.
